# Magnesium (Mg) and Neurodegeneration: A Comprehensive Overview of Studies on Mg Levels in Biological Specimens in Humans Affected Some Neurodegenerative Disorders with an Update on Therapy and Clinical Trials Supplemented with Selected Animal Studies

**DOI:** 10.3390/ijms252312595

**Published:** 2024-11-23

**Authors:** Agnieszka Ścibior, Juan Llopis, Paweł P. Dobrakowski, Tomasz Męcik-Kronenberg

**Affiliations:** 1 Laboratory of Oxidative Stress, Department of Biomedicine and Environmental Research, Institute of Biological Sciences, Faculty of Medicine, The John Paul II Catholic University of Lublin, Konstantynów St. 1J, 20-708 Lublin, Poland; 2 Department of Physiology, Institute of Nutrition and Food Technology “José Mataix”, Biomedical Research Centre, University of Granada, 18100 Armilla, Spain; jllopis@ugr.es; 3 Sport and Health Research Centre, University of Granada, 18016 Granada, Spain; 4 Psychology Institute, Humanitas University in Sosnowiec, Jana Kilińskiego St. 43, 41-200 Sosnowiec, Poland; paweldobrakowski@interia.pl; 5 Department of Pathomorphology, Faculty of Medical Sciences in Zabrze, Medical University of Silesia, 3 Maja St. 13, 41-800 Zabrze, Poland; patolog@interia.pl; 6 Collegium Medicum im. Dr Władysław Biegański, Jan Długosz University, Washington St. 4/8, 42-200 Częstochowa, Poland

**Keywords:** magnesium, neurodegenerative disorders, animal and human studies, body fluids, brain

## Abstract

Neurodegenerative diseases, characterized by neuron loss, are a group of neurological disorders that adversely affect the lives of millions of people worldwide. Although several medicines have been approved for managing neurodegenerative diseases, new therapies allowing for a significant slowdown in the progression of neurodegenerative syndromes are constantly being sought. Magnesium (Mg), a crucial mineral necessary for the functioning of organisms, is important to normal central nervous system (CNS) activity. Although the effects of this bioelement on the CNS are relatively well recognized, its role in the pathophysiology of neurological disorders in humans is not yet well characterized. Therefore, the main goal of this review is to collect data about a possible association between Mg and neurodegenerative disorders such as Alzheimer’s disease (AD), Parkinson’s Disease (PD), and Amyotrophic lateral sclerosis (ALS) in humans. Hence, the levels of Mg in blood, cerebrospinal fluid (CSF), urine, and hair from subjects with AD, PD, and ALS are compiled to detect possible variations in the levels of this mineral in the biological specimens of people with neurodegenerative illnesses. Additionally, the findings from an animal model are summarized to offer the reader a deeper insight into studies on Mg in the context of neuroprotection and neurodegeneration. Data provided in the present review indicate that Mg, due to its neuroprotective, antioxidant, anti-inflammatory, and mitochondrial-supportive properties, could be a potential therapeutic agent for AD, PD, and ALS. However, more epidemiological studies with standardized methods of dietary assessment and Mg measurement are necessary to recognize its exact role in neurodegenerative disorders. Moreover, extensive well-designed clinical trials are also needed to establish definitive therapeutic protocols and optimal dosages, and to ensure long-term safety of this mineral supplementation in AD, PD, and ALS patients.

## 1. Introduction

The present review is an attempt to provide thorough knowledge on the influence of magnesium (Mg) on neurodegeneration in humans. After the Introduction section, in which the main goals are formulated, the review comprises a few main sections and subsections. Section 2, containing five subsections (2.1–2.5), provides information about the search strategy with two flowcharts of the literature review process. One of them refers to humans and the other one is focused on animals. Section 3, with three subsections (3.1–3.3), enriched by a historical framework related to Mg, provides a concise summary of basic information about this mineral and its biological role. Section 4 briefly summarizes symptoms of Mg deficiency, mainly focusing on the nervous system. Section 5 provides an overview of findings from studies on the levels of Mg in the central nervous system (CNS) of animals exposed to a high level of metals involved in neurodegeneration. Section 6, composed of two subsections (6.1 and 6.2), compiles data on the concentration of Mg in biological specimens from subjects with Alzheimer’s disease (AD), Parkinson’s disease (PD), and Amyotrophic Lateral Sclerosis (ALS). Section 7, which contains six subsections (7.1–7.6), summarizes data on the neuroprotective mechanisms of Mg in AD, PD, and ALS, collects information about the neuroprotective efficacy of this bioelement in animals following experimental brain injury, and provides a summarizing note from studies on animals and humans on the neuroprotective efficacy of Mg in the context of cognitive function. Section 8 describes the role of Mg in the brain. Section 9, comprising three subsections (9.1–9.3), provides a concise overview of the therapeutic effects of Mg in humans with AD, PD, and ALS and compiles information about Mg in clinical trials for humans with AD, PD, and ALS. Finally, Section 10 provides the conclusions.

One of the main goals of this review is to collect concise knowledge of the impact of Mg on neurodegeneration in humans. It is important to provide objective information about a possible association between Mg and neurodegenerative disorders such as AD, PD, and ALS in humans. To achieve this, we have tried to collect evidence on the possible disturbance of Mg patterns in biological fluids and CNS tissues of humans with AD, PD, and ALS. Hence, a reliable analysis of literature data has been carried out and selected literature results from studies of the levels of this mineral in human biological specimens have been overviewed and illustrated in an accessible form to anyone interested in neurodegeneration and Mg in general. Moreover, as Mg plays a crucial role in maintaining brain health, our focus was directed towards data, obtained from an animal model, related to the neuroprotective efficacy of this bioelement and possible changes in the levels of this mineral in the CNS of animals exposed to metals involved in neurodegenerative disorders. This has allows us to offer the reader a deeper insight into studies on Mg in the context of neuroprotection and neurodegeneration. Additionally, the present review examines the literature concerning therapeutic effects of Mg in humans with AD, PD, and ALS and clinical trials of this mineral for AD, PD, and ALS diseases. Finally, a brief historical framework related to Mg has been provided to draw the reader’s attention to the important events associated with this essential bioelement in terms of time.

## 2. Methodology—Literature Search Strategy

### 2.1. Databases: General Outline

The literature search in English-language databases (i.e., PubMed, Scopus, and Web of Science) was conducted to collect relevant data. Only research articles and abstracts written in English were reviewed. In the case of articles with an unavailable full text, we corresponded with the authors in an attempt to obtain the full article. In the absence of a reply, the information provided in the abstracts was included in the current paper. Additionally, the reference lists of selected articles collected from the above-mentioned databases were manually reviewed to identify additional records (i.e., full-text papers or abstracts) that were potentially relevant to the topic.

### 2.2. Query Terms Used for the Literature Search on Magnesium in Neurodegenerative Disorders in Humans

The search was focused on the ‘Title’ and ‘Abstract’ and keywords such as ‘magnesium’, ‘Alzheimer’s disease’, ‘Parkinson’s Disease’, ‘Amyotrophic lateral sclerosis’, ‘neurodegeneration’, ‘dementia’, ‘neurodegenerative diseases’, ‘brain’, ‘cerebrospinal fluid’, ‘blood’, tissue’, ‘hair’, ‘urine’, and ‘humans’ were searched in various combinations to obtain the records on the levels of magnesium in biological specimens, i.e., in the brain, cerebrospinal fluid (CSF), blood, urine, and hair of patients with neurodegenerative illnesses such as Alzheimer’s disease (AD), Parkinson’s Disease (PD), and Amyotrophic lateral sclerosis (ALS).

### 2.3. Query Terms Used for the Literature Search on Magnesium in the Central Nervous System (CNS) of Animals Exposed to Metals Involved in Neurodegeneration

The search terms, focused on the ‘Title’ and ‘Abstract’, which included terms such as ‘magnesium’, ‘metals’, ‘central nervous system’ (CNS), ‘animals’, ‘brain’, ‘tissue’, ‘neurological diseases’, ‘amyotrophic lateral sclerosis’, and ‘Alzheimer’s disease’, were used to obtain records limited to the possible changes in the levels of magnesium in CNS of animals exposed to metals involved in neurodegeneration.

### 2.4. Search Results and Literature Review Flowchart on Magnesium in Neurodegenerative Disorders in Humans

**Figure 1:**
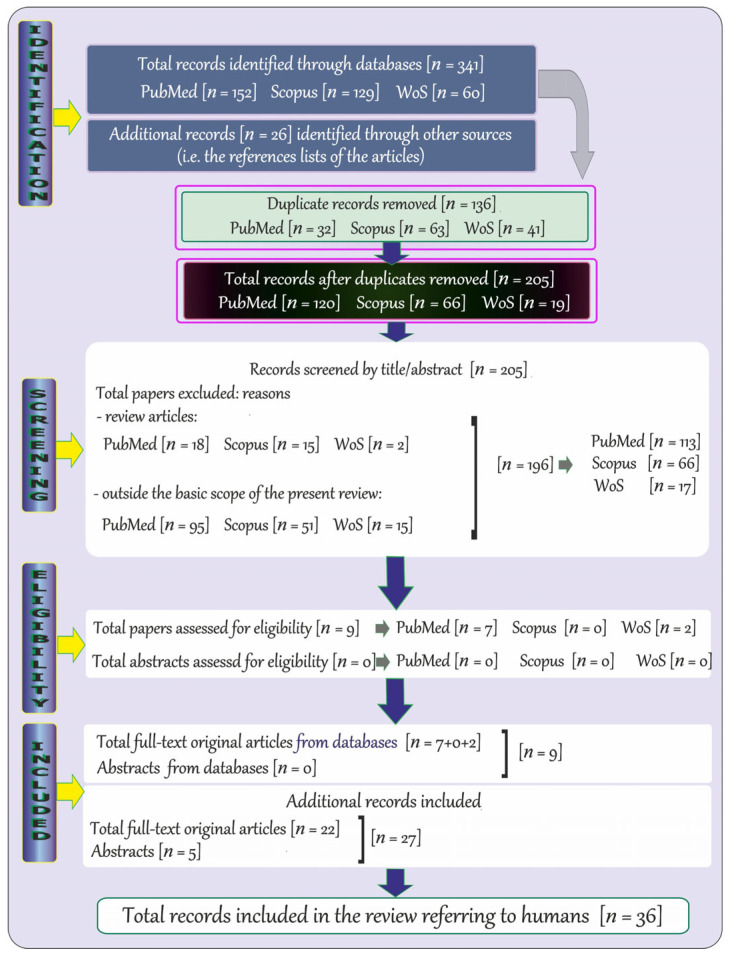
Flowchart of the systematic literature review on the levels of magnesium in biological specimens of humans with certain neurodegenerative disorders.

The adopted strategy of searching for specific keywords allowed us to detect records in PubMed, Scopus, and WoS that are relevant to the topic of Mg in some neurodegenerative diseases in humans. The flowchart provided below (Figure 1) shows the process employed to identify records on Mg in certain neurodegenerative illnesses in humans.

A total of 341 records published in English were identified through the databases listed above, i.e., 152 through PubMed, 129 through Scopus, and 60 through WoS. After removal of 136 duplicate items (PubMed: 32, Scopus: 63, and WoS: 41), the remaining records (*n* = 205; PubMed: 120, Scopus: 66, and WoS: 19) were initially screened by title and abstract. Afterwards, review articles and those that did not address the topic were excluded (PubMed: 113, Scopus: 66, and WoS: 17). Next, a total of 9 potentially relevant full-text articles (PubMed: 7 and WoS: 2) were further examined. Moreover, 27 additional records (i.e., 22 full-text original articles and 5 abstracts) relevant to the topic were included by manual review of bibliographies. Finally, a total of 36 records (i.e., 31 full-text original papers and 5 abstracts) were included in the current review. 

### 2.5. Search Results and Literature Review Flowchart on Magnesium in the Central Nervous System (CNS) of Animals Exposed to Metals Involved in Neurodegeneration

**Figure 2:**
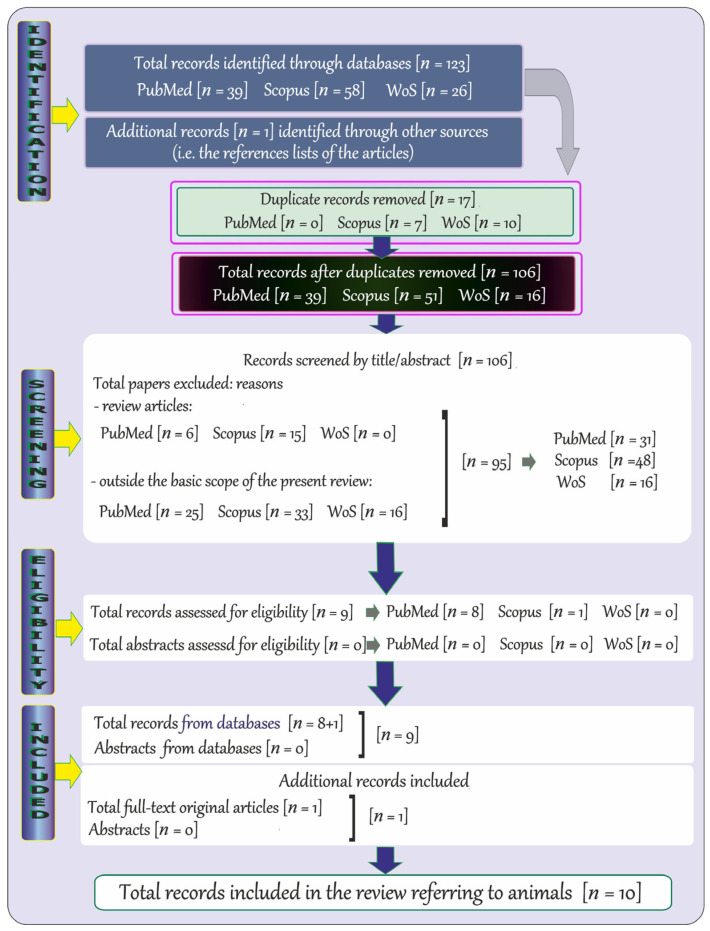
Flowchart of the systematic literature review on the possible changes in the levels of magnesium in central nervous system (CNS) of animals exposed to metals involved in neurodegeneration.

The adopted strategy of searching for specific key terms also allowed us to detect records relevant to the topic of the possible changes in the levels of Mg in CNS of animals exposed to metals involved in neurodegeneration. The flowchart (Figure 2) shows the steps of the methodology described in this subsection.

At the beginning, a total of 123 records published in English were revealed through the databases, i.e., 39 through PubMed, 58 through Scopus, and 26 through WoS. Next, a total of 17 duplicate records (PubMed: 0, Scopus: 7, and WoS: 10) were removed, and the remaining items (*n* = 106; PubMed: 39, Scopus: 51, and WoS: 16) were initially screened by the titles and abstracts. Afterwards, review articles (*n* = 21; PubMed: 6, Scopus: 15, and WoS: 0) and records that were far beyond the topic addressed in the present review were excluded (*n* = 74; PubMed: 25, Scopus: 33, and WoS: 16), and 9 potentially relevant items were further examined (PubMed: 8, Scopus: 1, and WoS: 0). Finally, a total of 9 records from databases and 1 additional record relevant to the topic (included by manual review of bibliographies) were included (10 in total). 

## 3. Magnesium: Selected Issues in a Nutshell

### 3.1. Backgroud

Magnesium (oxidation state 2+, Mg^2+^) is a divalent metal belonging to the second group of the periodic table of the elements, i.e., the beryllium group. It frequently occurs as a free cation in aqueous solutions or as the mineral part of a substantial variety of compounds [1]. Ions of this metal have the ability to form chelate complexes and are characterized by high biochemical activity [1,2]. 

Magnesium is the fourth most abundant mineral in the human body, after calcium (Ca), sodium (Na), and potassium (K), and after potassium, it is the second most important intracellular cation necessary for the proper growth and development of the human body [3]. The content of this mineral in the organism is physiologically regulated through such mechanisms as intestinal absorption, renal reabsorption and excretion, and exchange from the body pool, i.e., from bones [1,4].

### 3.2. Biological Role of Magnesium

Magnesium is an important ion with respect to various biological functions [5]. It serves as a cofactor for about 600 cellular enzymes and activates approximately 300 enzymes involved in many physiological processes [4]. This bioelement takes part in protein biosynthesis, DNA and RNA synthesis, systemic mineral metabolism, nerve transmission, muscle contractility, neuromuscular conduction, vasomotor tone, glucose and insulin metabolism, regulation of blood pressure, and thermoregulation, among other physiological processes. Mg also plays an important role in the homeostasis of elements such as Na, K, and Ca and is involved in the metabolism of adenosine triphosphate (ATP) and bone mineralization. By participating in the metabolism of proteins and nucleic acids, it acts as a regulator of the process of cell division and growth; by forming complexes with phospholipids of biological membranes, it is responsible for their stability and permeability. Given its many functions, Mg plays a major role in disease prevention and overall health [2]. 

### 3.3. A Brief Historical Framework Related to Magnesium

**Figure 3:**
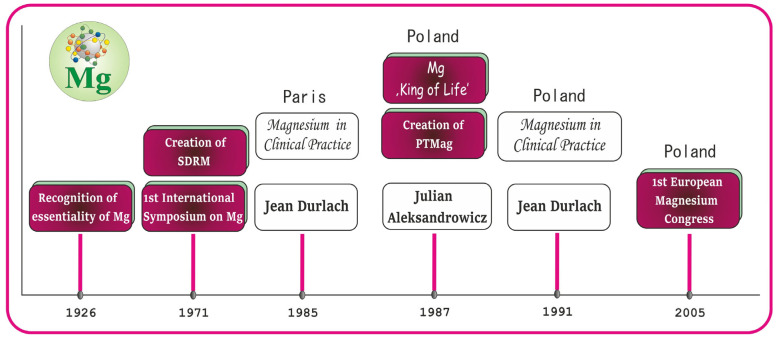
Summary of selected issues on magnesium (Mg) on the timeline: a historical view. Elaborated on the basis of available literature data [6,7,8,9].

A brief historical view of Mg is summarized in the timeline in Figure 3 to draw attention to some important events associated with this essential nutrient. As illustrated, in 1926, the essential character of Mg was acknowledged [6]. Forty-five years later, in 1971, the First International Symposium on Magnesium was organized and the international Society for the Development of Research on Magnesium (SDRM), which promotes the publication of Mg books and Mg journals, was created [7,8]. Fourteen years after this event, i.e., 1985, Professor Jean Durlach (a French scholar) summarized the state of knowledge about Mg at that time in an extensive monograph entitled *Le Magnésium en Pratique Clinique*, which was published in many countries, including in Poland in 1991. In Poland, the initiator of the studies on Mg was Professor Julian Aleksandrowicz, who emphasized the unique role of this bioelement in the human body, calling it the ‘King of life’. In 1987, Professor Julian Aleksandrowicz led to the establishment of the Polish Society for Magnesium Research (PTMag in Polish) and became its first chairman [9]. The cooperation between Professor Julian Aleksandrowicz and Professor Jean Durlach resulted in the organization of the first European Magnesium Congress in Cracow (Poland) in 2005.

## 4. Magnesium Deficiency—Nervous System Symptoms: A Brief Outline

**Figure 4:**
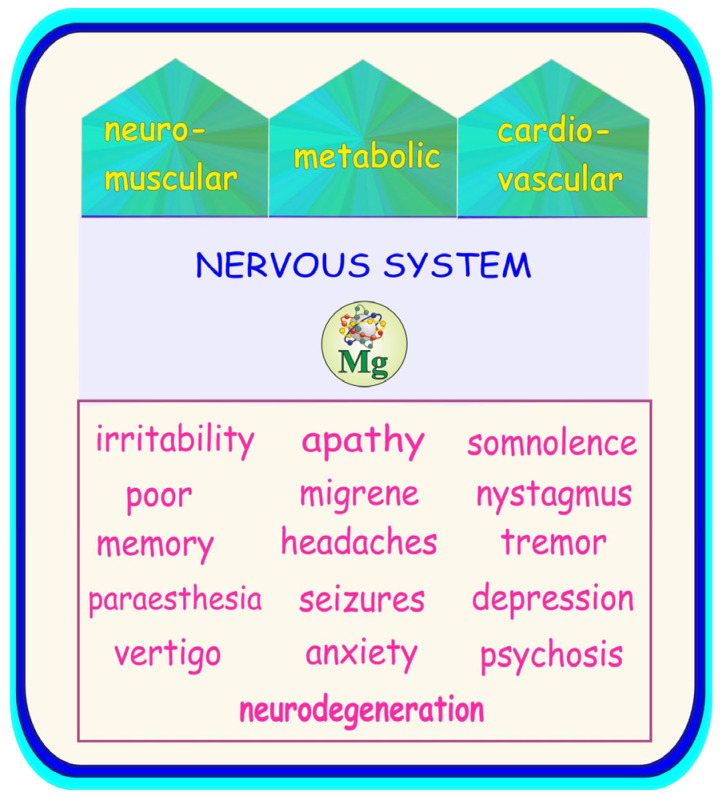
Summary of symptoms of magnesium (Mg) deficiency including the nervous system. Elaborated on the basis of available literature data [2,5,10,11,12,13,14,15,16,17,18].

As mentioned in the previous section, Mg is an essential mineral that is required for numerous biochemical, cellular, and physiological processes [3]. Deficiency in this key nutritional macroelement can result in multiple symptoms, including those in the nervous system, which are briefly summarized in Figure 4. As presented, a Mg deficit most often manifests as disturbances within the neuromuscular and cardiovascular systems. Metabolic disorders may also occur [10,11,12]. 

The symptoms of deficiency of this mineral in the nervous system include irritability, apathy, somnolence, nystagmus, seizures, migraine headaches, vertigo, poor memory, paraesthesia, anxiety, depression, and psychosis [1,3,5,13,14,16,17,19]. Moreover, it has been reported that Mg deficit may be involved in the pathogenesis of certain neurodegenerative diseases, including Alzheimer’s disease (AD) [15,18,20], the most widespread cause of dementia [5]. 

## 5. Magnesium in CNS: A Summarizing Note from Studies on Animals Exposed to an Unbalanced Mg Diet and/or a High Level of Metals Involved in the Neurodegeneration

As some metals have been reported to be associated with neurodegeneration and may affect the concentration of Mg in the CNS, we summarized studies on the levels of this bioelement in the CNS of animals exposed to high levels of certain elements related to neurodegenerative disorders. Our attention has been drawn to the involvement of aluminum (Al) and fluorine (F) in neurodegenerative processes. Additionally, our attention has been drawn to reports on the effect of an unbalanced Mg diet with a high content of Al on the level of this bioelement in the CNS tissues. Details concerning these studies conducted in a rodent model are listed in Table 1 and described in the current section. The impact of other neurodegenerative disease metals, such as zinc (Zn) and copper (Cu), has not been included because there is a lack of papers in the literature that would describe the effect of these elements on the level of Mg in biological specimens of people with neurodegenerative disorders such as AD, PD, or ALS.

The whole brain and some different brain structures, such as the cortex, cerebellum, brainstem, and hippocampus, as well as the pons and spinal cord were used to determine the concentration of Mg in CNS. The research material mainly originated from rats and, to a much lesser extent, from mice. In terms of the number of studies that have been conducted so far in this research area, only ten papers were found in the literature [21,22,23,24,25,26,27,28,29,30].

One of the studies on the impact of high levels of metals associated with neurodegeneration on the levels of Mg in the CNS of animals was performed by Lubkowska and co-workers [30]. These authors determined the concentration of Mg in the cerebellum, cortex, and hippocampus of male and female rats exposed to aluminum (Al), which is a widely acknowledged neurotoxin that can accumulate in the CNS and cause cognitive deficiency and dementia [31]. The metal was administered separately and in combination with fluorine (F), which also participates in neurodegenerative processes [32], in drinking water for 31 days. The researchers did not observe statistically significant changes in the level of Mg in the investigated brain structures. No statistically significant alterations in the concentration of Mg in the cerebellum, cortex, hippocampus, and brainstem of male mice receiving Al (100 mg/kg body weight) for 2 months were noted by Yang et al. [28]. Yang and Wong [29] reported no changes in the concentration of this mineral in the cerebellum, cortex, hippocampus, or brainstem of male mice receiving brick tea liquid with high Al content for 1 and 2 months. Similarly, no significant changes in the content of Mg were observed by Yasui et al. [22] in the cerebellum, the occipital and frontal cortex, or the pons of male rats fed a low Ca-Mg diet with high Al content for 90 days; however, a statistically significant decline in the level of this element occurred in the spinal cord (SP). A decrease in the concentration of Mg in the SP of rats receiving a diet with high Al content for 60 or 90 days was also noted by Yasui and Ota [27] and Yasui et al. [25,26]. Additionally, in a previous study conducted by Yasui’s research group [21], a significant decrease in the level of Mg in the SP of rats fed an unbalanced Ca-Mg diet with high Al content was observed. Moreover, Kihira et al. [33] reported that a low Ca-Mg and high-Al diet provided to mice for 11–31 months induced loss of neurons and tau-related neuronal degeneration. Neuronal loss with axonal swellings was also noted by Yoshida et al. [34] in experimental animals fed a Ca- and Mg-deficient diet with added Al. Based on the findings obtained by Kihira et al. [33] and Yoshida et al. [34] as well as the results summarized in Table 1, which point to a decrease in the level of Mg in some CNS tissues, it can be suggested that chronic exposure to low Ca-Mg conditions and an elevated concentration of Al, which can inhibit the activity of Mg-requiring enzymes or impair Mg transport [35], may induce Mg imbalance and neuronal degeneration, thereby increasing the risk of development of neurodegenerative disorders. As speculated by some researchers, who studied the relationship between metal behavior and degenerative processes in CNS tissues of Mg-deprived and Al-supplemented animals [36], Mg depletion may accelerate the uptake of Al into the brain, which may lead to the development of degenerative processes.

**Table 1:** Summary of data on the levels of Mg in CNS of animals exposed to an unbalanced Mg diet and/or high concentration of metals involved in the neurodegeneration.

Metals	Doses	Unit	Route	Time	Animals	Mg					Ref.
						CB	Cortex		HP	BS	
F	100	ppm	dw	31 d	W-rats ♂	→	→		→	nd	[30]
Al	300	ppm	dw	31 d	W-rats ♂	→	→		→	nd	
F	100	ppm	dw	31 d	W-rats ♀	→	→		→	nd	
Al	300	ppm	dw	31 d	W-rats ♀	→	→		→	nd	
F + Al	100 + 300	ppm ppm	dw	31 d	W-rats ♂	→	→		→	nd	
F + Al	100 + 300	ppm ppm	dw	31 d	W-rats ♀	→	→		→	nd	
Al * Al *	1 1	% %	*per os* *per os*	1 mo 2 mo	Mice ♂ Mice ♂	→ →	→ →		→ →	→ →	[29]
Al	100	mg/kg b.wt.	dw	2 mo	Mice ♂	→	→		→	→	[28]
						**CB**	**FC**	**OC**	**Pons**	**SP**	**Ref.**
Ca-Mg + Al	3-2 194	mg/100 g mg/100 g	with diet	90 d	W-rats ♂	→	→	→	→	↓	[22]
Al + Ca	194 3	mg/100 g mg/100 g	with diet	60 d	W-rats ♂	nd	nd		nd	↓	[27]
Ca-Mg + Al	Low ^§^ High ^§^	n/a n/a	with diet	90 d	W-rats ♂	nd	nd		nd	↓	[25,26]
						**CNS tissues**				**SP**	**Ref.**
Ca-Mg + Al	Low ^§^ High ^§^	n/a n/a	with diet	n/a	Rats	→				↓	[21]

F: fluorine; AL: aluminum; dw: drinking water; d: days: mo: months; B.wt.: body weight; W-Rats: Wistar rats; CB: cerebellum; BS: brainstem; HP: hippocampus; FC: frontal cortex; OC: occipital cortex; SP: spinal cord; CBS: central nervous system; nd: not determined; n/a: not available. →: without statistically significant changes; ↓: statistically significant decrease; *: liquor of brick tea contained a high Al content. ^§^ based on the information provided in the abstract only.

One notable study was the experiment conducted by Oyanagi and co-workers [37] in simulated conditions of human life in Guam, where high incidences of Parkinsonism-dementia complex (PDC) and ALS were noted. As low levels of Ca and Mg and high levels of Al are thought to be implicated in the pathogenesis of these diseases in Guam, the experiment was performed on rats exposed to low Ca and/or Mg intake over two generations. The results of this study showed significant losses of dopaminergic neurons in the substantia nigra in 1-year-old animals with consistently low Mg intake. Based on these findings, the authors suggested that low Mg intake over generations may be involved in the pathogenesis of substantia nigra degeneration in humans. Another animal experiment which aimed to test the hypothesis that catalepsy, behavioral immobility, and Parkinson’s disease-related symptoms result from functionally impaired dopaminergic neurons in mice fed a low-Ca/Mg diet showed that these animals developed catalepsy (resulting from hypofunctioning of dopaminergic neurons) after 3 weeks on a low-Ca/Mg diet. This supported the hypothesis that low Ca/Mg intake may be one of the etiological factors in neurodegenerative disorders.

It has also been described that rats fed a diet deficient in Mg (200 mg/kg feed) can exhibit signs of hyperexcitability, behavioral alterations, neurological impairments leading to an increase in vigilance states, decreases in sleep time and quality [38], and death preceded by generalized convulsions; however, no changes have been observed in the content of Mg [23,24], Zn [39] and Ca and P [24] in the whole brain.

## 6. Magnesium in Biological Specimens of Humans with Neurodegenerative Illnesses

### 6.1. Magnesium in the Brain and Cerebrospinal Fluid of Humans with Alzheimer’s Disease (AD), Parkinson’s Disease (PD), and Amyotrophic Lateral Sclerosis (ALS): A Summarizing Note 

Summarized values of the concentrations of Mg in the different parts of the brain and in CSF (cerebrospinal fluid) of people with neurodegenerative disorders are listed in Table 2.

Studies conducted by Gerhardsson et al. [40], Boström et al. [41,42], Hozumi et al. [43], Jouini et al. [45], and Babić Leko [44], who examined metal changes (including Mg) in the CSF of subjects with neurodegenerative disorders, did not indicate any significant differences in the level of Mg in the CSF of AD patients compared to the control [Table 2]. As for Parkinson’s disease, studies conducted by Forte et al. [54], Bocca et al. [53], and Hozumi et al. [43] did not indicate significant alterations in the concentration of this bioelement in the CSF of PD patients compared to the control. However, Bocca et al. [53], who aimed to obtain data about mineral concentrations (including Mg) in the CSF of a large population of PD patients, compared to the control, in order to evaluate whether alterations in their levels could be either a cofactor of risk for developing PD or a marker of the disease, found that the Mg level in the CSF of Parkinsonians decreased with the duration and severity of the disease. In turn, Hozumi et al. [43], who measured the levels of Mg in patients with ALS, found markedly elevated concentrations of this mineral in the CSF of people with ALS (by 21 %) compared to the control. In this case, the number of control subjects used in the study was relatively small (i.e., 15) and the control subjects were markedly younger (~48 yr) than the patients with ALS (65 yr) (Table 2). As emphasized, the increased level of Mg in ALS may be pathognomonic features. 

Regarding the CNS regions, there are few papers presenting changes in the level of Mg in the brain of AD, PD, and ALS subjects. For example, Andrasi et al. [46,47], who investigated the concentrations of Mg in various brain areas in AD patients, found a statistically significant decrease in the level of this mineral in AD patients in comparison with the control. For example, in the Ammon’s horn (AH), cortex entorhinalis (CE), and globus pallidus (GP)m, the content of this mineral in AD subjects was about 18%, 19%, and 12% lower, respectively, compared to the control. A decrease in the concentration of Mg in the brain was also found by Barbiroli et al. [52], who performed in vivo phosphorus magnetic resonance spectroscopy on the occipital lobes (OLs) of 15 patients with multiple system atrophy, 13 patients with idiopathic PD, and 16 age-matched healthy subjects. In this case, the level of this bioelement in the OL of PD cases turned out to be about 21% lower in comparison with that noted in the control individuals. Further, Uitti et al. [49], who studied four anatomical brain areas, i.e., the frontal cortex (FC), caudate nucleus (CN), substantia nigra (SN), and cerebellum (CB) in 9 PD brains and 12 control brains and measured the concentrations of 24 metals, including Mg, to identify any abnormalities in metal levels specific to PD also found a statistically significant decrease in the concentration of Mg in the CN of the brains of patients with Parkinson’s disease. A statistically significant decrease in the concentration of Mg was also noted by Yasui and co-workers [50] in the cortex, white matter (WM), basal ganglia (BG), and brain stem (BS) of PD brains, compared to control brains. In turn, Scholefield et al. [51], who intended to compare the concentrations of nine essential metals across some different brain regions, i.e. the SN, cingulate gyrus (CG), locus coeruleus (LC), hippocampus (HP), primary visual cortex (PVC), middle temporal gyrus (MTG), primary motor cortex (MCX), cerebellum (CB), and medulla oblongata (MED) in cases of PD and controls, showed a statistically significant decrease in the concentration of Mg in brain structures such as the MCX and MED. As for ALS, Yasui et al. [48], who elucidated the involvement of metals as a factor in the pathogenesis of ALS on the Kii Peninsula of Japan (a well-known high-incidence area of ALS with low Mg and Ca content in soil and drinking water) and determined the levels of Mg and Ca in samples of CNS tissue taken from postmortem ALS cases, found statistically significantly reduced average levels of Mg in regions such as the internal capsule (IC), crus cerebri (CC), and spinal cord (SP) of individuals with ALS. Moreover, the mean value of the Mg level in the 26 examined brain regions (i.e., precentral gyrus, including the motor cortex, postcentral gyrus, frontal, parietal, temporal, occipital and cerebellar cortex, insula, internal capsule, crus cerebri, frontal, parietal, temporal, occipital and cerebellar white matter, thalamus, caudate nucleus, globus pallidus, putamen, substantia nigra, red nucleus, pons, olivary nucleus, medulla and spinal cord) in ALS cases was also found to be significantly lowered. Additionally, the Ca/Mg ratio in the CNS regions of ALS subjects was significantly elevated in comparison with the controls. Based on these findings, the authors suggested that there may be abnormalities in the metal/mineral metabolism of individuals with ALS. In summation, the decreased concentration of Mg noted by some authors in certain brain regions of AD, PD, and ALS patients [46,48,49,50,51,52] may point to a deficiency of this bioelement in the brain during degeneration.

### 6.2. Magnesium in the Blood, Urine, and Hair of Humans with Alzheimer’s Disease (AD), Parkinson’s Disease (PD), and Amyotrophic Lateral Sclerosis (ALS): A Summarizing Note 

Summarized values of the concentrations of Mg in the blood (including serum (S), plasma (PL), whole blood (WB), red blood cells (RBC), and white blood cells (WBC)) and in urine (U) and hair (H) are listed in Table 3.

Normal serum Mg levels typically range from 0.75 to 0.95 mmol/L, while in CSF, they range between 0.77 and 1.17 mmol/L [76]. As for the level of Mg in the blood (i.e., in the serum, plasma, RBC, and WBC) of patients with AD or PD listed in the above table, Zaken et al. [65], Shore et al. [61], Koc et al. [62], Babić Leko et al. [44], Gerhardsson et al. [40], Xu et al. [66], Boström et al. [42], Brackenridge and McDonald [69], Borella et al. [71], Gustaw-Rothenberg et al. [63], Alimonti et al. [64], and Bocca et al. [53] did not find statistically significant differences in the concentration of this element in the blood of subjects with AD or PD compared to the control. Forte et al. [54] and Bocca et al. [53] did not report any significant changes in the concentration of Mg in the urine of PD patients; likewise, Bocca et al. [53] and Koc et al. [62] reported no significant changes of this nature in the hair of PD subjects and in the hair of AD individuals, respectively. In turn, Shore et al. [61], Kobayashi et al. [72], and Forte et al. [74] demonstrated a statistically significant decrease in the concentration of Mg in the hair of AD and PD patients compared to the control. A statistically significant decrease in the concentration of Mg was also found by Bargagallo et al. [56], Balmus et al. [57], Singh et al. [58], Kurup and Kurup [60], and Cilliler et al. [59] in the serum of AD patients and Vural et al. [67], Lemke et al. [68], and Ahmed et al. [70] reported similar findings in the plasma of AD subjects. In turn, other authors, i.e., Forte et al. [54], Bocca et al. [53], and Anirudham et al. [73], noted a statistically significant increase in the concentration of Mg in the whole blood and serum of PD patients. The levels of this element in the PD individuals turned out to be about 9.5 %, 19.6 %, and 23% higher, respectively, in comparison with those noted by the authors in the control. As stressed by Forte et al. [54], the increased value of Mg found in the serum of PD patients may suggest an involuntary intake of this mineral as drug excipients during treatment of PD. Another noteworthy study was conducted by Kieboom and co-workers [15], who examined the levels of Mg in the serum and the relationship of this mineral with the risk of all-cause dementia and AD in a large prospective population-based cohort with long-term follow-up (a mean age of 64.9 yr; 56.6% were women; 823 participants were diagnosed with all-cause dementia). The authors revealed that both low and high serum Mg levels are associated with a greater risk of all-cause dementia. 

Taken together, as evident from the studies provided in Section 6.1 and Section 6.2, no significant changes were noted in the concentration of Mg in the CSF between the control and AD, PD, or ALS patients, except in one study with a large age difference between the control group and ALS subjects, in which a significant rise in the level of this mineral was observed. In contrast, the levels of this mineral in certain CNS tissues of individuals with AD, PD, and ALS were reported to be significantly lowered, suggesting that the deficit of Mg may be an important factor in the development of fatal neurodegenerative disorders.

Further, some studies summarized in this section demonstrated that the concentration of Mg in the blood of AD individuals significantly decreased, whereas the concentration of this mineral significantly increased in PD patients. Moreover, the decrease in the concentration of Mg in the blood of AD patients intensified with the severity of the disease. These data may suggest a potential role of Mg in the etiopathogenesis of AD and PD and point to a specific Mg pattern which can characterize AD and Parkinsonism. It was also found, in a systematic review and meta-analysis, that serum and plasma levels of Mg were significantly reduced in AD patients compared with healthy controls, which could indicate that Mg deficiency may be a risk factor of AD and Mg supplementation may be a potentially valuable adjunctive treatment for AD [77]. However, other studies did not indicate any significant alterations in the level of Mg in the blood of both AD and PD subjects. Therefore, further investigations with a larger number of participants and standardized ways to measure Mg levels with an assessment of intracellular free Mg should established to improve our understanding of the findings. It also cannot be excluded that such research may help with the use of Mg in the serum/plasma as a potential biomarker of progression/severity of AD/PD. Moreover, taking into account the results of a study conducted by Miyake et al. [78], who examined the relationship between Mg consumption and the risk of PD in non-Western populations and demonstrated that a higher intake of this bioelement was independently associated with a reduced risk of PD, as well as the study of Powers et al. [79], who examined the associations of PD with dietary nutrients, including minerals, vitamins, and fats (a case–control study in the Seattle area) and found no relationship between intake of Mg and PD, further epidemiological studies with standardized methods of dietary assessment are also required to better recognize the exact role of Mg in neurodegenerative disorders. Additionally, there is a need for well-designed and carefully controlled clinical trials involving Mg in AD, PD, and ALS patients; such trials could provide more valuable information and clarify whether supplementation with Mg could be an adjunctive treatment in these neurodegenerative diseases. Details on this issue are provided in the further part of this review.

## 7. Magnesium: Neuroprotection

### 7.1. Neuroprotective Mechanisms of Magnesium Action: A Summarizing Note

Despite the fact that the exact mechanism of the neuroprotective effect of Mg has not been fully recognized, the following hypotheses include anti-oxidative effects [80,81], anti-inflammatory effects [82,83], anti-apoptotic effects [84], blocking of *n*-methyl-D-aspartate receptors (NMDA-R) [81,85], inhibition of L-type calcium channels [82], membrane stabilization [86,87], and cerebral blood flow modulation [88]. 

### 7.2. Mechanisms of Action of Magnesium in Alzheimer’s Disease (AD)

AD, which is a public health issue, is the main cause of dementia that affects most areas of the brain. At present, there is no cure for dementia, and the development of pharmacological treatments has been unsuccessful for the past 30 years; thus, it has been suggested that greater attention should be directed towards prevention [89].

Magnesium has garnered interest for its potential therapeutic effects in AD due to its role in numerous biochemical processes in the brain. While the precise mechanisms by which Mg might influence AD pathology are still being elucidated, several key research topics have been highlighted.

- Neuroprotection and synaptic function.

Magnesium is essential for maintaining synaptic plasticity and neuronal function. It helps regulate NMDA (*n*-methyl-D-aspartate) receptors, which are crucial for synaptic plasticity, learning, and memory [90]. Dysregulation of these receptors is a hallmark of AD. This element also plays a role in reducing neuronal excitotoxicity, which is caused by excessive stimulation of neurons leading to cell damage or death, a process implicated in AD progression [91].

- Oxidative stress reduction.

Magnesium has antioxidant properties that can help reduce oxidative stress, a condition characterized by excessive free radicals that damage cells, which is closely associated with AD pathology [92]. By enhancing antioxidant defenses, this mineral may protect neurons from oxidative damage.

- Anti-inflammatory effects.

Inflammation is a critical component of AD pathology [93]. Magnesium can modulate inflammatory responses in the brain, potentially reducing the neuroinflammation associated with AD [94].

- Amyloid beta and tau pathology.

Magnesium may influence the aggregation and clearance of amyloid-beta (Aβ) peptides, which form plaques that are a hallmark of AD [95]. It might also affect the phosphorylation of tau protein, another key pathological feature of AD. Proper Mg levels may help reduce abnormal tau phosphorylation, thereby preventing the formation of neurofibrillary tangles [96].

- Mitochondrial function.

Magnesium supports mitochondrial function and energy metabolism in neurons [97]. Dysfunctional mitochondria contribute to AD progression, so maintaining mitochondrial health through adequate Mg levels might have protective effects [98].

### 7.3. Mechanisms of Action of Magnesium in Parkinson’s Disease (PD)

PD is a brain condition that causes problems with movement, mental health, and other health issues. It results from a complex combination of various pathological events, including abnormal protein aggregation, altered lysosome function, mitochondrial dysfunction, endoplasmic stress, glutaminergic excitotoxicity, oxidative stress, and neuroinflammation. These all promote neuronal death, targeting the dopaminergic neurons of the basal ganglia in particular [99]. Currently, PD cannot be cured, but medications can help control the symptoms.

Magnesium has attracted interest for its potential therapeutic effects in PD. The mechanisms by which this element might benefit individuals with PD are diverse and involve several key aspects of neuronal health and function.

- Neuroprotection.

Magnesium has neuroprotective properties that can help safeguard neurons against damage. It can mitigate excitotoxicity, which occurs when neurons are overactivated by excitatory neurotransmitters, leading to cell damage or death. It also helps maintain the integrity of the blood–brain barrier (BBB), protecting the brain from harmful substances [20].

- Dopaminergic neuron support.

Magnesium is involved in the regulation of dopamine synthesis and release. Proper levels of this element can support the function of dopaminergic neurons, which are particularly affected in PD patients [100].

- Reduction of oxidative stress.

Oxidative stress, resulting from an imbalance between free radicals and antioxidants, plays a significant role in PD pathogenesis [101]. Mg has antioxidant properties that help reduce oxidative damage in neurons.

- Anti-inflammatory effects.

Chronic neuroinflammation is a hallmark of PD. Mg can modulate inflammatory pathways, potentially reducing neuroinflammation and slowing disease progression [102].

- Mitochondrial function.

Mitochondrial dysfunction is implicated in PD [103]. Mg supports mitochondrial health and energy production, factors which are critical for the survival and function of neurons.

- Metal ion homeostasis

Dysregulation of metal ions, such as iron, is involved in PD pathology. Mg can influence metal ion homeostasis, potentially reducing the harmful effects of metal ion imbalance in the brain [104].

### 7.4. Mechanisms of Action of Magnesium in Amyotrophic Lateral Sclerosis (ALS)

ALS is a progressive neurodegenerative disease that affects motor neurons, leading to muscle weakness and atrophy. While there is currently no cure for ALS, research has explored various potential therapeutic approaches, including the use of Mg. This element is known to play several roles in the central nervous system, and its potential therapeutic effects in ALS are based on its neuroprotective, antioxidant, and anti-inflammatory properties. Some of the key mechanisms and findings related to magnesium’s potential effects on ALS are listed below.

- Neuroprotective effects—excitotoxicity reduction.

Magnesium acts as a natural antagonist of NMDA (*n*-methyl-D-aspartate) receptors, which are involved in excitotoxicity. Excitotoxicity, caused by excessive glutamate activity, leads to neuronal injury and death and is implicated in ALS. By blocking excessive NMDA receptor activity, Mg can help protect motor neurons from excitotoxic damage [90].

- Calcium homeostasis.

Magnesium helps regulate the influx of calcium (Ca) into neurons. Dysregulated Ca homeostasis is a feature of ALS pathology, contributing to neuronal degeneration. Magnesium’s ability to stabilize Ca levels can therefore be neuroprotective [20].

- Reduction of oxidative stress.

Oxidative stress, resulting from an imbalance between the production of reactive oxygen species (ROS) and the body’s ability to detoxify them, is a significant factor in ALS [105]. Mg has antioxidant properties that help neutralize ROS, thereby protecting neurons from oxidative damage [106].

- Modulation of inflammatory responses.

Chronic neuroinflammation is a hallmark of ALS [107]. Mg can modulate inflammatory pathways and reduce the production of pro-inflammatory cytokines, potentially mitigating the inflammatory damage to motor neurons.

- Support of mitochondrial health

Mitochondrial dysfunction is implicated in the pathology of ALS [108]. Mg plays a role in maintaining mitochondrial function and energy production. Ensuring proper mitochondrial health is crucial for neuron survival and function.

**Figure 5:**
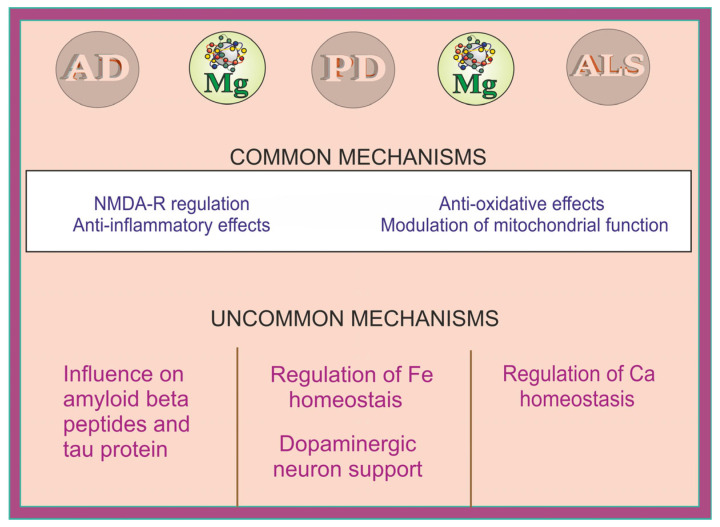
Summary of the neuroprotective mechanisms of magnesium (Mg) involved in AD, PD, and ALS. AD: Alzheimer disease; PD: Parkinson disease; ALS: Amyotrophic lateral sclerosis.

The mechanisms by which Mg may affect the pathology of AD, PD, and ALS are graphically summarized below (Figure 5).

### 7.5. Neuroprotective Efficacy of Magnesium—Experimental Brain Injury: A Summarizing Note from Studies in an Animal Model 

**Figure 6:**
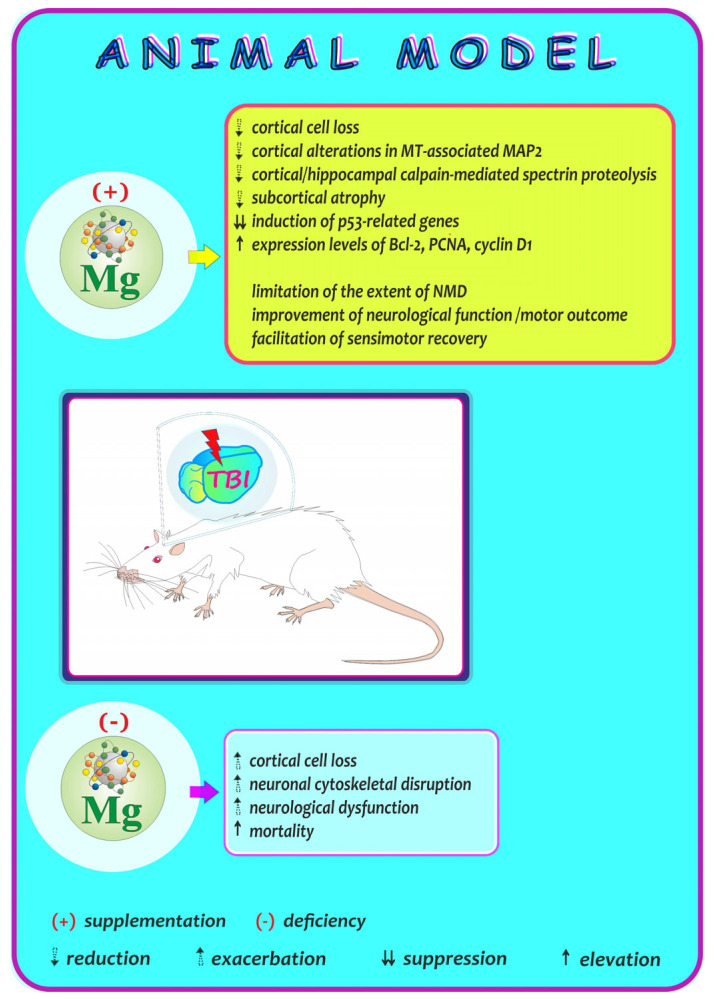
Summary of data on the impact of magnesium (Mg) following an experimental traumatic brain injury (TBI) in an animal model. MT: microtubule; MAP2: MT-associated protein; Bcl-2: anti-apoptotic protein; PCNA: proliferating cell nuclear antigen; NMD: neurological motor dysfunction. Elaborated on the basis of available literature cited in Section 7.5.

As magnesium is involved in the maintenance of the homeostasis of all the tissues, including the brain, in this section, we summarize studies on the neuroprotective efficacy of this bioelement in animals following an experimental traumatic brain injury (TBI) [109,110,111,112,113,114,115,116,117] (Figure 6). One of the works that fit into this issue is a study conducted by McIntosh’s research group [110], who examined the influence of treatment with Mg on post-traumatic neurological outcomes following brain injury in rats. The authors found that the administration of Mg in the form of magnesium chloride (MgCl_2_) at 30 min following TBI can effectively limit the extent of neurological motor dysfunction. In turn, Hoane et al. [114], who investigated whether Mg therapy could effectively limit behavioral impairments after cortical injury in rats given regimens of MgCl_2_ ending 24 h before surgery, indicated that pre-treatment with MgCl_2_ prevents the subcortical atrophy of striatal neurons following cortical injury, thereby resulting in behavioral improvement in the Mg-treated rats. Based on these findings, the authors concluded that daily supplementation with Mg may provide protection against some deleterious effects of brain injury, which, as highlighted, may be particularly important for subjects at an increased risk of head injuries, e.g., those competing in contact sports. Another research group, whose aim was to examine whether post-traumatic administration of MgCl_2_ can restore the Mg concentration and thus initiate neurologic recovery [115], demonstrated that treatment with Mg reduced post-traumatic neuromotor impairments but failed to improve learning ability in brain-injured rats. Further, the evaluation of post-traumatic cognitive outcomes (performed using a modification of the Morris Water Maze technique) conducted by Smith et al. [111] revealed a significant attenuation of post-traumatic memory dysfunction in rats treated with MgCl_2_. Other researchers also demonstrated that post-traumatic administration of Mg in the form of MgCl_2_ or magnesium sulfate (MgSO_4_) attenuates brain edema and improves the neurological outcome in a model of experimental TBI [112,113]. In turn, a study conducted by Lee and co-workers [117] (undertaken to characterize the molecular and cellular mechanisms underlying the neuroprotective effects of Mg) explored whether post-injury treatment with Mg (as MgCl_2_) alters the cell survival and the expression of related proteins after brain injury in rats. It showed that post-treatment with Mg suppresses the induction of p53-related genes (especially Bax) and elevates the expression of cell survival-related proteins, such as Bcl-2, proliferating cell nuclear antigen (PCNA), and cyclin D1. As stressed by the authors, the findings of their study provide a molecular basis for the efficiency of Mg in treating TBI-induced tissue damage.

It is also worth mentioning the study conducted by McIntosh and co-workers [109], who evaluated the relationship between the decline in Mg and neurological outcomes following TBI. The authors showed that Mg deficiency (after dietary restriction) results in a decrease in the level of Mg in the brain, exacerbates neurological dysfunction, and increases the mortality of rats following brain injury, whereas pre-treatment with Mg in the form of MgSO_4_ 15 min before brain injury prevents the decrease in this bioelement in the brain and improves neurological function. As emphasized by the authors, Mg pre-treatment has a beneficial effect on post-traumatic neurologic outcomes following TBI in rats. In turn, exacerbation of cortical cell loss and cytoskeletal alterations within the cortex and hippocampus in Mg-deficient rats after TBI was noted by Saatman et al. [116]. In a model of TBI, these investigators showed that post-administration of Mg effectively attenuates cortical cell loss, cortical alterations in microtubule (MT)-associated protein-2 (MAP-2), and both cortical and hippocampal calpain-mediated spectrin proteolysis. Based on these data, they suggested that cortical cell death and cytoskeletal disruption in cortical and hippocampal neurons may be sensitive to the Mg status after experimental brain injury and may be mediated in part through modulation of calpains.

### 7.6. Neuroprotective Efficacy of Magnesium in the Context of Cognitive Function: A Summarizing Note from Studies on Animals and Humans 

Mg is known to play a significant role in cognitive function [118]. Research revealed that this bioelement is fundamental to the functioning of NMDA receptors involved in memory processing [119]. Detailed information about the neuroprotective efficacy of Mg in the context of cognitive function are concisely summarized below. For example, the studies conducted by Slutsky and co-workers [120] in a rodent model in which young (2-month-old), aging (12- to 18-month-olds), and aged rats (22- to 24-month-olds) were used demonstrated that the increase in the concentration of Mg in the brain after the treatment with this mineral resulted in enhancements in learning abilities, working memory, and short- and long-term memory. Another study performed by Tai et al. [121], who investigated the efficacy of Mg in the form of magnesium lithospermate B (MLB) against lipopolysaccharide (LPS)-induced neurodegeneration in LPS-injected mice, demonstrated that the administration of MLB ameliorates LPS-induced neurodegeneration and microglial activation in the hippocampus, which appears to be crucial for long-term episodic memory [122], in adult animals. In turn, Bardgett et al. [123], who intended to characterize the behavioral consequences of Mg deficiency in mice, found that a chronic reduction in dietary Mg impairs memory. Specifically, mice fed a consistently Mg-deficient diet demonstrated significant memory deficits in both contextual and cued conditioning tests [123].

During the literature review, an article was also found which described a human study aimed at investigating associations between the dietary mineral intake and the risk of mild cognitive impairment and other mild cognitive disorders in cognitively healthy individuals. The study showed that the increased Mg intake decreases the risk of cognitive impairment [124]. Another study, which aimed at examining whether higher intake of potassium, calcium, and Mg is able to reduce the risk of dementia in a general population of Japanese elderly adults, revealed that dietary intakes of K, Ca, and Mg were associated with a lower risk of all-cause dementia in the general Japanese elderly population [125]. However, as stressed by the authors, plausible mechanisms to account for these associations remain unclear. 

Further, Xu et al. [96] found that the administration of Mg increased the brain Mg levels and prevented learning and memory impairments in streptozotocin-induced sporadic AD model rats. The authors suggested that treatment with Mg at an early stage may decrease the risk of cognitive impairment in AD [96]. Another in vivo study, in which a gelatin/polyvinyl alcohol-loaded Mg hydroxide nanocomposite was used, showed the protective effect of the formulated Mg compound on the cognitive and synaptic impairments in AD-induced rats [126]. In turn, Miyake et al. [78], who investigated the relationship between metal consumption and the risk of PD in Japan using data from a multicenter hospital-based case–control study, showed that higher intake of Mg is associated with a reduced risk of PD and that the inverse association remained after adjustment for intake of other elements. 

## 8. Magnesium in the Brain

Mg is fundamental for nerve signal transmission and the maintenance of ionic homeostasis [20]. This mineral controls the influx of Ca by regulating Ca voltage-dependent channels, while intracellularly, it inhibits Ca release from cytosolic stores through inositol 1,4,5-trisphosphate and ryanodine receptors [127]. It is an agonist of the ionotropic gamma aminobutyric acid type A receptor (GABA_A_-R) [128]. 

In early development, GABA_A_-R signaling prompts Mg release from the mitochondria, and the increase in cytoplasmic Mg activates mTOR, which facilitates the formation of neural networks [129]. 

Mg inhibits the glutamate *n*-methyl-D-aspartate receptor (NMDA-R) at the physiological membrane potential, which is around − 70 mV, when glutamate only acts on the α-amino-3-hydroxyl-5-methyl-4-isoxazole-propionate (AMPA) receptor, thereby preventing sustained stimulation of NMDA-R, which leads to neuronal death. The protective action of Mg is also due to its ability to block the opening of the mitochondrial permeability transition pore and the subsequent release of cytochrome c, which culminates in apoptosis [130]. 

Mg also protects the integrity and function of the BBB, the impenetrable cellular scaffold that regulates CNS homeostasis, and therefore, maintains healthy neurological function [131,132].

## 9. Magnesium in Therapy of Some Neurodegenerative Diseases and Clinical Trials 

### 9.1. Alzheimer’s Disease (AD): A Summarizing Note

Clinical studies on Mg supplementation in AD patients have shown discrepant results, but there are some promising findings.

- Cognitive function.

Some studies suggest that Mg supplementation can improve cognitive function in AD patients. Cognitive function studies mainly focus on magnesium-L-threonate. It is this form that effectively crosses the BBB. In a group of healthy Chinese people, an improvement in cognitive function was observed after 30 days of supplementation. The effects were particularly pronounced in the oldest participants. It should be noted, however, that the preparation used also contained vitamin D3, vitamin C, vitamin B6, and phosphatidylserine. The possible additive effect of the above substances must be taken into account [133]. In the clinical trial NCT02210286, which involved people diagnosed with AD, L-threonate administered for 2 months did not improve or stabilize the tested cognitive functions.

An interesting study was conducted on a small group of elderly people who reported memory problems [134]. As additional necessary inclusion criteria, increased anxiety level and sleep disorders were used. This triad significantly indicates neurodegenerative disorders [135,136]. Improvement was found in all examined cognitive domains in patients who met the criteria for MCI (mild cognitive impairment). The authors emphasized that this improvement was not observed in every participant in the study. This highlights the need to indicate a biomarker of therapy success.

- Biomarker changes.

Changes in cerebrospinal fluid biomarkers related to AD pathology (e.g., Aβ and tau proteins) have been observed in some studies following Mg supplementation, indicating potential biochemical effects. The effects of Mg on Aβ aggregation remain disputable, as some studies show that this element reduces Aβ plaques while others report an elevation of Aβ plaques following Mg treatment [137]. Recently it has been described that treatment with magnesium orotate decreases the production of AB in SH-SY5Y-APP695 cells [138].

- Brain volume.

Clear patterns of gray matter loss in mostly hippocampal and temporal regions were found across all clinical stages in patients with AD [139]. A study of more than 6000 cognitively healthy participants found that people who consume more than 550 milligrams of Mg each day have a larger brain volume (gray matter, hippocampus) compared with a normal Mg intake of about 350 milligrams a day. This research highlights the potential benefits of a high-Mg diet and the role it plays in promoting good brain health [140].

In another study, an elevated serum Mg level was associated with greater brain volumes and lower odds of subclinical cerebrovascular disease, suggesting that Mg has beneficial effects on pathways related to neurodegeneration and cerebrovascular damage. These data are important in the context of AD, because in some patients we are dealing with mixed dementia with a vascular component [141].

### 9.2. Parkinson’s Disease (PD): A Summarizing Note

Clinical trials exploring the effects of Mg in PD are less extensive compared to those investigating other neurodegenerative diseases. The association between circulating Mg and PD remains ambiguous and controversial. A meta-analysis showed that the serum Mg levels in PD cases were significantly higher than those in healthy controls. In addition, higher CSF Mg levels were observed in patients with PD in comparison with the normal range [142]. The presence of low levels of Mg in brain tissue and high levels of Mg in CSF and serum support the possibility of dysfunctional Mg transporters in PD.

### 9.3. Amyotrophic Lateral Sclerosis (ALS): A Summarizing Note

While the theoretical benefits of Mg in ALS are compelling, clinical evidence on this topic remains limited. The relation between dietary intake of this element and ALS risk was explored in five large prospective cohort studies. Dietary Mg intake was not associated with ALS risk. This finding does not support a protective effect of this mineral intake on ALS risk [143]. 

## 10. Conclusions 

On the basis of the data included in the present review, we may conclude that more epidemiological studies with standardized methods of dietary assessment and Mg measurement are crucial for recognizing the exact role of this mineral in neurodegenerative illnesses. It seems that regular monitoring of the levels of Mg and appropriate supplementation can be important elements in the prevention and therapy of these diseases. However, further studies are necessary to evaluate the potential benefits of supplementation with this mineral in the prevention and treatment of neurodegenerative disorders. The current data are insufficient to make unambiguous recommendations. It is worth mentioning that choosing proper Mg salt that more efficiently traverses the BBB to be utilized in case of Mg deficiency to harmonize the levels of Mg in the brain is important. As for Mg deficiency, a deficit of this mineral increases neuroinflammation by altering gut microbiota, which influences the nervous system through the activation of the vagal nerve, the production of cytokines, and the release of neuropeptides and neurotransmitters. It is possible that low Mg intake unbalances the gut microbiota–brain axis.

It is not without significance that maintaining the proper Mg concentration through a balanced diet may support cognitive function and overall brain health. Adequate levels of this mineral may play a protective role, and studies on the metabolism of Mg may help with the development of new therapies to support the treatment and prevention of neurodegenerative illnesses. Numerous clinical studies are also needed to confirm the clinical application of this bioelement.

As far as Alzheimer’s disease is concerned, Mg is a promising potential therapeutic agent for AD due to its neuroprotective, antioxidant, anti-inflammatory, and mitochondrial-supportive properties. While preclinical studies and some clinical trials have shown beneficial effects, more research is needed to establish definitive therapeutic protocols, optimal dosages, and long-term safety of Mg supplementation in AD patients. The known mechanisms of magnesium’s action on the central nervous system seem to be important for the prevention of neurodegeneration. However, no therapeutic effect on AD has been demonstrated so far.

As for Parkinson’s disease, Mg also shows promise as a potential therapeutic agent for PD due to its neuroprotective, antioxidant, anti-inflammatory, and mitochondrial-supportive properties. Briefly, human research related to Mg concentrations in PD is severely lacking, despite the growing amount of evidence implicating Mg in animal studies. Therefore, there is a need for more studies in PD patients focusing on Mg concentrations in order to get a better consensus on the relationship between Mg and PD.

As far as Amyotrophic Lateral Sclerosis is concerned, limited clinical trials have been conducted, and the results are not conclusive. Some trials have shown modest benefits, while others have not demonstrated significant improvement. In summary, while Mg shows potential due to its various beneficial properties, its use in treating ALS is not yet established. For now, Mg can be considered only as part of a broader, multimodal approach to managing ALS.

## Figures and Tables

**Table 2:** Summary of the results of the levels of magnesium (Mg) in brain and cerebrospinal fluid in humans with AD, PD, and ALS.

Subjects	Number	Age (Mean/*Median*/*Ranges*)	Concentration (Mean/*Median*) *	Biological Fluids/Tissues	Units	Ref.
AD cases						
Control AD	*n* = 54 *n* = 173	73 (60–94) 75 (52–86)	*28* (25–37) *28* (23–36)	CSF CSF	mg/L mg/L	[40]
Control AD	*n* = 49 *n* = 159	73.1 75.4	28.4 (25.2–37.0) 27.7 (23.4–35.5)	CSF CSF	mg/L mg/L	[41]
Control AD	*n* = 49 *n* = 174	*73* (60–97) *74* (52–86)	*28.0* (25.2–37.0) *27.5* (23.4–35.5)	CSF CSF	mg/L mg/L	[42]
Control AD	*n* = 15 *n* = 21	48.4 65.4	29.6 31.8	CSF CSF	µg/mL µg/mL	[43]
Control AD	*n* = 19 *n* = 126	*61* (52–75) *72* (65–78)	27.8 30.2	CSF CSF	mg/L mg/L	[44]
Control AD	*n* = 53 *n* = 104	68.574 70.538	1.1502 1.130	CSF CSF	mmol/L mmol/L	[45]
Control AD	*n* = 20 *n* = 9	70 n/a	n/a n/a ↓	VBA (10 parts) VBA (10 parts)	n/a n/a	[46]
Control AD	*n* = 3 *n* = 3	61 77	680 666 606 673 628 557 ↓ 540 ↓ 625 623 552 ↓	AM CE CFP CFB GP AM CE CFP CFB GP	µg/g dw µg/g dw µg/g dw µg/g dw µg/g dw µg/g dw µg/g dw µg/g dw µg/g dw µg/g dw	[47]
ALS cases						
Control ALS	*n* = 1 *n* = 1 *n* = 1 *n* = 1 *n* = 1 Average Average Average Average Average *n* = 1 *n* = 1 *n* = 1 *n* = 1 *n* = 1 Average Average Average Average Average	69 65 69 75 74 37 61 67 61 55	389/681 519/553 591 393/606 484/437 512 471/612 587/639 559 330/650 529/567 518 460/665 461/483 574 409 643 516 536 552 339/566 435/378 417 217/156 321/230 238 277/287 298/366 283 567/522 472/423 489 311/352 303/384 323 342 377 ↓ 366 ↓ 356 ↓ 350 ↓	PG/IC CC/SP 26 CNS regions PG/IC CC/SP 26 CNS regions PG/IC CC/SP 26 CNS regions PG/IC CC/SP 26 CNS regions PG/IC CC/SP 26 CNS regions PG IC CC SP 26 CNS regions PG/IC CC/SP 26 CNS regions PG/IC CC/SP 26 CNS regions PG/IC CC/SP 26 CNS regions PG/IC CC/SP 26 CNS regions PG/IC CC/SP 26 CNS regions PG IC CC SP 26 CNS regions	µg/g dw µg/g dw µg/g dw µg/g dw µg/g dw µg/g dw µg/g dw µg/g dw µg/g dw µg/g dw µg/g dw µg/g dw µg/g dw µg/g dw µg/g dw µg/g dw µg/g dw µg/g dw µg/g dw µg/g dw µg/g dw µg/g dw µg/g dw µg/g dw µg/g dw µg/g dw µg/g dw µg/g dw µg/g dw µg/g dw µg/g dw µg/g dw µg/g dw µg/g dw µg/g dw µg/g dw µg/g dw µg/g dw µg/g dw µg/g dw	[48]
Control ALS	*n* = 15 *n* = 52	48.4 65	29.6 35.9 ↑	CSF CSF	µg/mL µg/mL	[43]
PD cases						
Control PD	*n* = 12 *n* = 9	70 73	539 530 452 560 482 471 ↓ 414 515	FC CN SN CB FC CN SN CB	µg/g dw µg/g dw µg/g dw µg/g dw µg/g dw µg/g dw µg/g dw µg/g dw	[49]
Control PD	n/a n/a	n/a n/a	n/a n/a n/a n/a n/a ↓ n/a ↓ n/a ↓ n/a ↓	Cotrex WM BG BS Cotrex WM BG BS	n/a n/a n/a n/a n/a n/a n/a n/a	[50]
Control PD Control PD Control PD Control PD Control PD Control PD Control PD Control PD Control PD	*n* = 9 *n* = 9 *n* = 27 *n* = 9 *n* = 9 *n* = 9 *n* = 9 *n* = 9 *n* = 27 *n* = 9 *n* = 9 *n* = 9 *n* = 9 *n* = 27 *n* = 9 *n* = 26 *n* = 9 *n* = 9	*87* (79–95) *78* (66–93)	20 (19.3–20.7) 19.7 (18.8–20.6) 22.8 (22.0–23.7) 22.2 (21.1–23.3) 19.4 (18.6–20.2) 18.7 (17.8–19.6) 20.4 (19.7–21.0) 19.6 (19.1–20.2) ↓ 26.7 (25.4–27.9) 24.8 (22.9–26.8) 24.2 (22.4–26.0) 22.9 (22.1–23.6) 25.3 (23.1–27.5) 24.2 (22.3–26.0) 26.4 (17.6–35.2) 20.7 (18.7–22.7) ↓ 20.1 (18.5–21.7) 20.7 (18.4–23.0)	SN SN CG CG LC LC MED MED HP HP PVC PVC MTG MTG MCX MCX CB CB	mmol/kg dw mmol/kg dw mmol/kg dw mmol/kg dw mmol/kg dw mmol/kg dw mmol/kg dw mmol/kg dw mmol/kg dw mmol/kg dw mmol/kg dw mmol/kg dw mmol/kg dw mmol/kg dw mmol/kg dw mmol/kg dw mmol/kg dw mmol/kg dw	[51]
Control PD	*n* = 16 *n* = 13	58 (42–76) 67 (47–75)	181 143 ↓	OL OL	mmol mmol	[52]
Control PD	*n* = 18 *n* = 91	63.3 65.5	n/a n/a →	CSF CSF	µg/mL µg/mL	[53]
Control PD	*n* = 15 *n* = 20	48.4 68.7	29.6 31.6	CSF CSF	µg/mL µg/mL	[43]
Control PD	*n* = 13 *n* = 26	54 65	21.229 20.913	CSF CSF	µg/L µg/L	[54]
Control AD	*n* = 28 *n* = 24	n/a n/a	n/a n/a	CSF CSF	n/a n/a	[55]

* All values are given as reported in the cited reports. *n*: number of patients; dw: dry weight; n/a: not available. AD: Alzheimer’s disease, PD: Parkinson’s Disease; ALS: amyotrophic lateral sclerosis; CSF: cerebrospinal fluid; AM: Ammon’s horn; CE: cortex entorhinalis; CFP: cortex frontalis basalis; GP: globus pallidus; FC: frontal cortex; CN: caudate nucleus; SN: substantia nigra; CG: cingulate gyrus; LC: locus coeruleus; MED: medula oblongata; PVC: primary visual cortex; MTG: middle temporal gyrus; MCX: primary motor cortex; WM: white matter; HP: hippocampus; CB: cerebellum; PG: precentral gyrus; BG: basal ganglia; BS: brain stem; IC: internal capsule; CC: crus cerebri; SP: spinal cord; CNS: central nervous system; OL: occipital lobe; VBA: various brain areas. ↓ Statistically significant decrease. ↑ Statistically significant increase. →: Not changed, compared to the control.

**Table 3:** Summary of the results of the levels of magnesium (Mg) in the blood, urine, and hair of humans with neurodegenerative diseases.

Subjects	Number	Age (Mean/*Meidian*/Ranges)	Concentration (Mean/*Median*/Ranges) *	Biological Fluids/Other	Units	Ref.
AD cases						
Control AD	*n* = 65 *n* = 36	73.8 73	0.53 0.50 ↓	serum serum	mmol/L mmol/L	[56]
Control AD	*n* = 15 *n* = 15	62.5 65.8	1316.46 994.08 ↓	serum serum	μg/dL μg/dL	[57]
Control AD	*n* = 100 *n* = 100	59.71 62.74	2.21 1.88 ↓	serum serum	mg/dL mg/dL	[58]
Control AD Control AD	*n* = 34 *n* = 37 *n* = 34 *n* = 37	n/a n/a n/a n/a	2.227 2.197 ^††^ 2.227 2.110 ^†††^ ↓	serum serum serum serum	mg/dL mg/dL mg/dL mg/dL	[59]
Control AD	*n* = 15 *n* = 15	*50*–*70* *50*–*70*	2.40 1.76 ↓	serum serum	mg/dL mg/dL	[60]
Control AD	*n* = 10 *n* = 10	62 63.7	1.83 1.89	serum serum	mEq/L mEq/L	[61]
Control AD	*n* = 33 *n* = 44	73.18 77.69	16.01 17.3	serum serum	μg/mL μg/mL	[62]
Control AD	*n* = 29 *n* = 30	65.4 69.1	1.9 2.43	serum serum	mg/dL mg/dL	[63]
Control AD	*n* = 124 *n* = 53	44.8 (20–84) 74.5 (58–86)	*17.424* *18.683*	serum serum	μg/L μg/L	[64]
Control AD ^†^	*n* = 42,698 *n* = 2761	72.05 78.88	2.065 2.064	serum serum	mg/dL mg/dL	[65]
Control AD	*n* = 14 *n* = 93	*61* (52–75) *72* (65–78)	24.1 24.3	plasma plasma	mg/L mg/L	[44]
Control AD	*n* = 54 *n* = 173	73 (60–94) 75 (52–86)	*21* (18–28) *21* (15–29)	plasma plasma	mg/L mg/L	[40]
Control AD	*n* = 43 *n* = 42	78.1 78.2	0.70 (0.67–0.71) 0.70 (0.69–0.73)	plasma plasma	mmol/L mmol/L	[66]
Control AD	*n* = 49 *n* = 174	*73* (60–97) *74* (52–86)	*21.1* (18.0–27.5) *21.3* (15.4–28.7)	plasma plasma	mg/L mg/L	[42]
Control AD Control AD	*n* = 50 *n* = 50 *n* = 50 *n* = 50	65.1 71.9 65.1 71.9	M: 2.12 1.91 ↓ F: 2.14 1.90 ↓	plasma plasma plasma plasma	mg/dL mg/dL mg/dL mg/dL	[67]
Control AD	*n* = 12 *n* = 12	75.2 77.5	0.70 0.58 ↓	plasma plasma	mmol/L mmol/L	[68]
Control AD	*n* = 26 *n* = 26	79.7 79.7	1.86 1.80	plasma plasma	mEq/L mEq/L	[69]
Control AD	*n* = 20 *n* = 20	*52.3* (47.2–68) *54* (48–81)	*0.9* (0.88–1.4) *0.3* (0.08–0.84) ↓	plasma plasma	mmol/L mmol/L	[70]
Control AD Control AD	n/a n/a n/a n/a	n/a n/a n/a n/a	n/a n/a → n/a n/a →	plasma plasma WBC WBC	n/a n/a n/a n/a	[71]
Control AD	*n* = 26 *n* = 26	79.7 79.7	12.83 13.33	RBC RBC	mEq/kg CS mEq/kg CS	[69]
Control AD	*n* = 28 *n* = 24	n/a n/a	n/a n/a	blood blood	n/a n/a	[55]
Control AD	*n* = 9 *n* = 9	62 63.7	60.9 37.4 ↓	Hair Hair	ppm ppm	[61]
Control AD	*n* = 33 *n* = 41	73.18 77.69	49 39	Hair Hair	μg/g μg/g	[62]
Control AD	n/a n/a	n/a n/a	n/a n/a ↓	Hair Hair	n/a n/a	[72]
PD cases						
Control PD	*n* = 32 *n* = 33	65 66	4.31 5.316 ↑	serum serum	µg/L µg/L	[73]
Control PD	*n* = 13 *n* = 26	54 65	17.491 19.163 ↑	serum serum	µg/L µg/L	[54]
Control PD	*n* = 124 *n* = 71	44.8 (20–84) 65.5 (41–81)	*17.424* *18.934*	serum serum	μg/L μg/L	[64]
Control PD	*n* = 18 *n* = 91	63.3 65.5	n/a n/a →	serum serum	µg/mL µg/mL	[53]
Control PD	*n* = 18 *n* = 91	63.3 65.5	26.5 31.7 ↑	blood blood	µg/mL µg/mL	[53]
Control PD	*n* = 13 *n* = 26	54 65	24.037 27.906	blood blood	µg/L µg/L	[54]
Control PD	*n* = 18 *n* = 91	63.3 65.5	n/a n/a →	urine urine	µg/mL µg/mL	[53]
Control PD	*n* = 13 *n* = 26	54 65	55.691 87.789	urine urine	µg/L µg/L	[54]
Control PD	*n* = 18 *n* = 91	63.3 65.5	43.3 36.4	hair hair	µg/g µg/g	[53]
Control PD	*n* = 17 *n* = 81	64.8 65.5	*43.3* *36.4* ↓	hair hair	µg/g µg/g	[74]
Others cases						
Guam C Guam P Guam P Guam P	*n* = 12 *n* = 12 *n* = 8/12 *n* = 5/12	49.7 (35–64) 61.8 (52–72) 61.8 (52–72) 61.8 (52–72)	*2.2* *90.0* *2.2* *83.0* ≤2.5 ↑ 26–60 ↓	serum urine serum urine serum urine	mg/dL mg/24 h mg/dL mg/24 h mg/dL mg/24 h	[75]

* All values are given as reported in the cited reports. *n*: number of patients; n/a: not available; hr: hours. AD: Alzheimer’s disease, ALS: amyotrophic lateral sclerosis; Guam C: Guamanian controls; Guam P: Guamanian patients; M: male; F: female; WBC: white blood cells; RBC: red blood cells; CS: cell solids. ↓: Statistically significant decrease; ↑: Statistically significant increase. →: Not changed, compared to the control. ↓: Lower but not statistically significant, compared to the control. ^†^: Alzheimer 43%. ^††^: patients evaluated as mild and moderate. ^†††^: patients evaluated as severe.

## Data Availability

Data sharing was not applicable.
